# Supplementary material for: Cytochrome P450 1B1 Expression Regulates Intracellular Iron Levels and Oxidative Stress in the Retinal Endothelium
Source: Int J Mol Sci. 2023 Jan 26;24(3):2420. doi: 10.3390/ijms24032420 (PMC9916835; doi:10.3390/ijms24032420)
Supplement: Supplementary file 1 [file ijms-24-02420-s001.zip › ijms-2149590-supplementary.pdf]

Supplementary Data

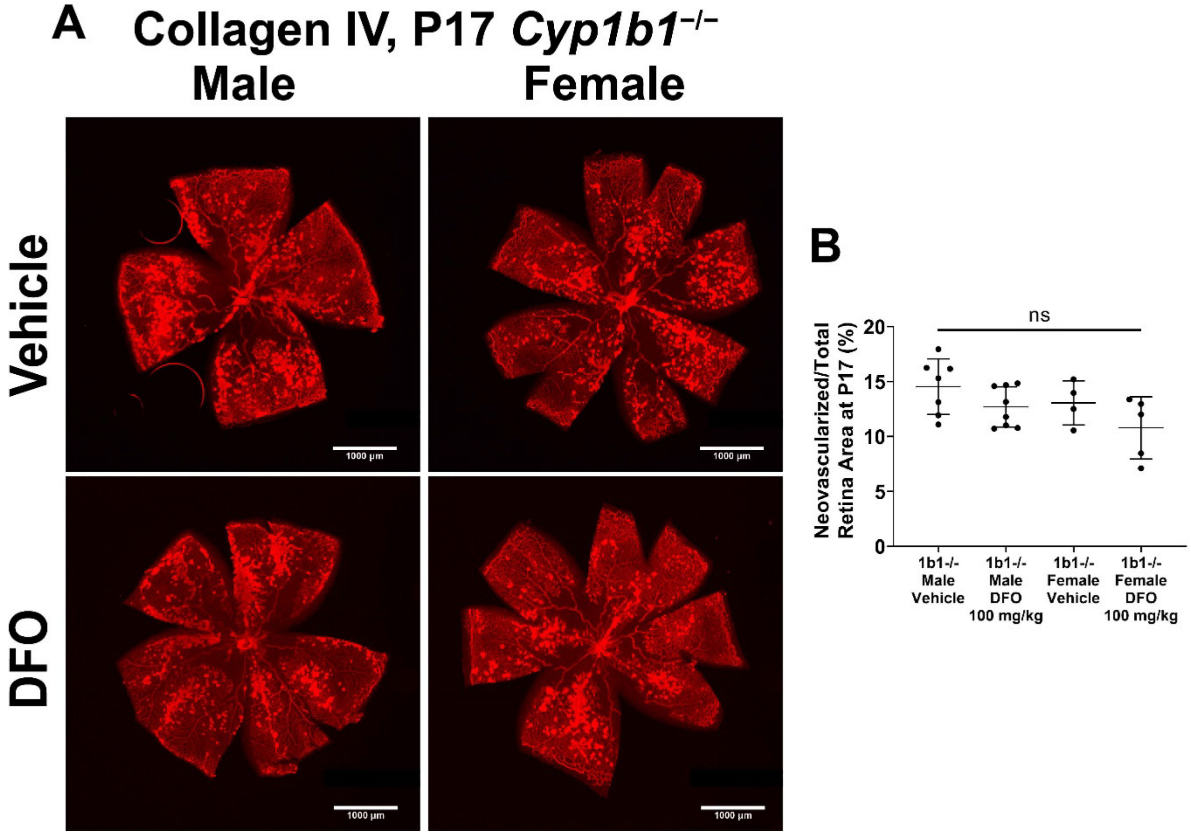

Figure S1. Iron chelation by DFO did not significantly restore neovascularization in *Cyp1b1*$^{-/-}$ mice during OIR. **(A)** Retinas from P17 *Cyp1b1*$^{-/-}$ mice that administrated with DFO (100 mg/kg, daily IP injection prepared in 50 µL saline, from P12-P16) during OIR were wholemount stained with anti-collagen IV antibody and imaged by fluorescent microscopy. Scale bars = 1000 µm. Quantitative assessment of the neovascularization was performed using ImageJ and percentages of the neovascularized area in the mice are shown in **(B)**. (ns: not significant, n ≥ 4; each point represents one retina).

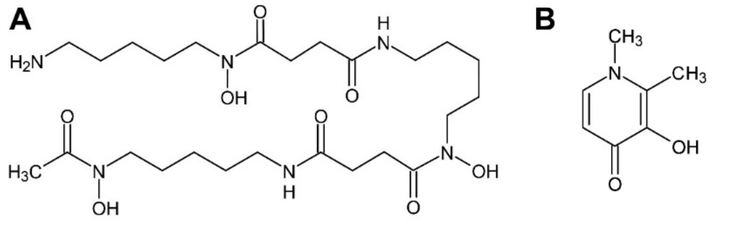

Deferoxamine **Deferiprone**

Figure S2. Chemical structure of iron chelators. Chemical structures of **(A)** deferoxamine and **(B)** deferiprone are shown.

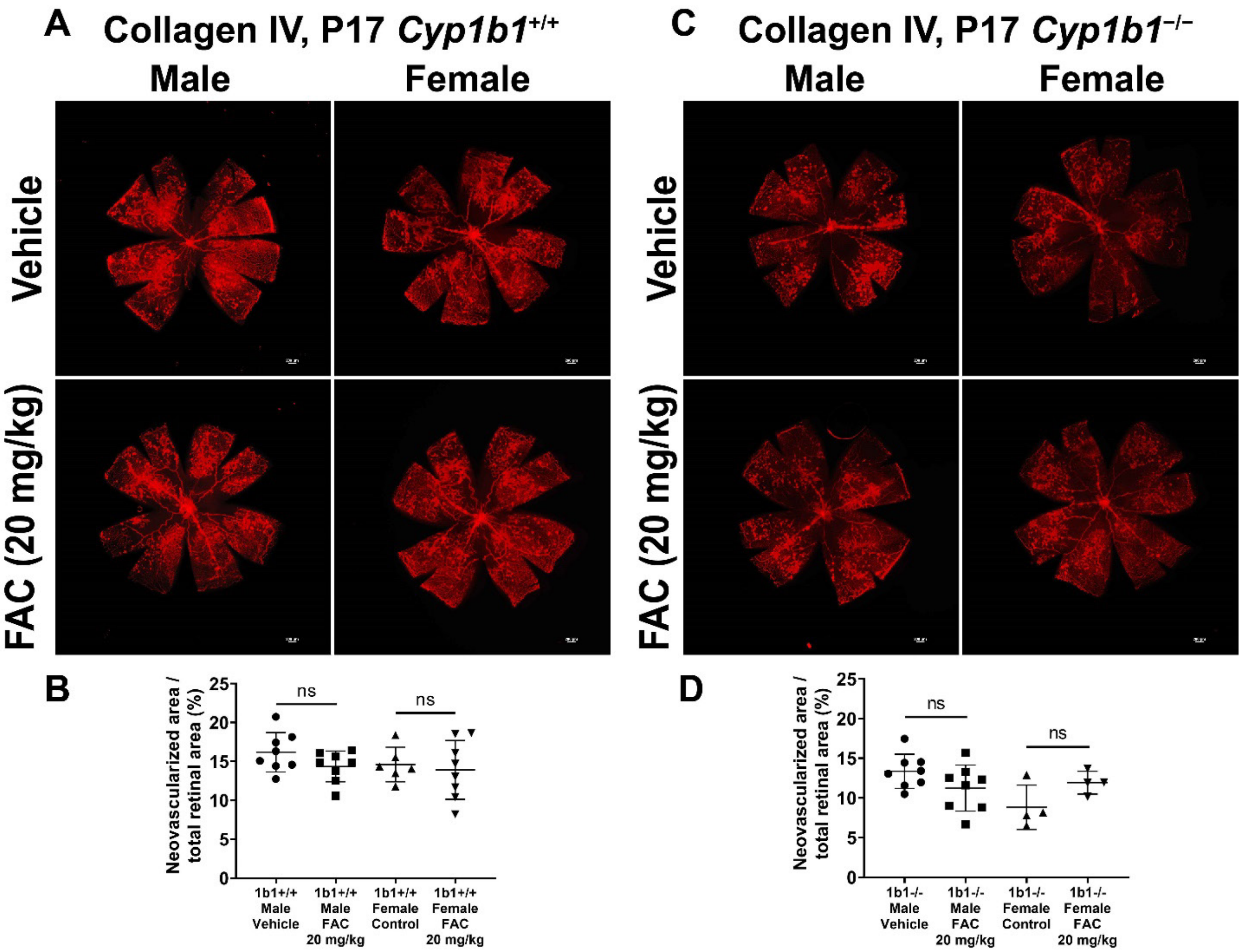

Figure S3. Iron supplementation with FAC did not alter the neovascularization in Cyp1b1$^{+/+}$ and Cyp1b1$^{-/-}$ mice during OIR. Retinas from P17 **(A)** *Cyp1b1$^{+/+}$* mice and **(C)** *Cyp1b1$^{-/-}$* mice that were administrated with FAC (20 mg/kg, daily IP injection prepared in 50 μL saline, from P12-P17) during OIR were wholemount stained with anti-collagen IV antibody and imaged by fluorescent microscopy. Scale bars = 1000 μm. Quantitative assessment of the neovascularization was performed using ImageJ and percentages of the neovascularized area in the mice are shown in **(B)** and **(D)** respectively (ns: not significant; n ≥ 4; each point represents one retina).
